# Hydrogen Assisted Cracking in Pearlitic Steel Rods: The Role of Residual Stresses Generated by Fatigue Precracking

**DOI:** 10.3390/ma10050485

**Published:** 2017-05-02

**Authors:** Jesús Toribio, Leticia Aguado, Miguel Lorenzo, Viktor Kharin

**Affiliations:** Fracture & Structural Integrity Research Group (FSIRG), University of Salamanca (USAL), E.P.S., Campus Viriato, 49022 Zamora, Spain; laguado@usal.es (L.A.); mlorenzo@usal.es (M.L.); gatogris@usal.es (V.K.)

**Keywords:** stress corrosion cracking, hydrogen assisted cracking, hydrogen assisted fracture, hydrogen embrittlement, hydrogen diffusion, residual stress field, pearlitic steel rods

## Abstract

Stress corrosion cracking (SCC) of metals is an issue of major concern in engineering since this phenomenon causes many catastrophic failures of structural components in aggressive environments. SCC is even more harmful under cathodic conditions promoting the phenomenon known as hydrogen assisted cracking (HAC), hydrogen assisted fracture (HAF) or hydrogen embrittlement (HE). A common way to assess the susceptibility of a given material to HAC, HAF or HE is to subject a cracked rod to a constant extension rate tension (CERT) test until it fractures in this harsh environment. This paper analyzes the influence of a residual stress field generated by fatigue precracking on the sample’s posterior susceptibility to HAC. To achieve this goal, numerical simulations were carried out of hydrogen diffusion assisted by the stress field. Firstly, a mechanical simulation of the fatigue precracking was developed for revealing the residual stress field after diverse cyclic loading scenarios and posterior stress field evolution during CERT loading. Afterwards, a simulation of hydrogen diffusion assisted by stress was carried out considering the residual stresses after fatigue and the superposed rising stresses caused by CERT loading. Results reveal the key role of the residual stress field after fatigue precracking in the HAC phenomena in cracked steel rods as well as the beneficial effect of compressive residual stress.

## 1. Introduction

Stress corrosion cracking (SCC) is one of the most frequent causes of failure of structural components in aggressive environments [1]. This phenomenon proceeds in different ways depending on the electrochemical conditions of the environment. Thus, for anodic conditions (above the hydrogen discharge line of H_2_O in a Pourbaix diagram for the system steel-H_2_O), a phenomenon known as *localized anodic dissolution* (LAD) takes place causing the loss of material by generating ferrous oxides [2]. Conversely, in the case of cathodic conditions (beneath the hydrogen discharge line of H_2_O in a Pourbaix diagram) the damage appears in a quite different way as the so-called *hydrogen assisted cracking* (HAC) [3]. In this case, the environment promotes the creation of atomic hydrogen that is adsorbed at the metal surface and later absorbed into the material. Later on, hydrogen is transported by diffusion [4,5,6,7] towards prospective damage areas. It then accumulates to a critical concentration associated with hydrogen assisted damage at the microstructural level. This process is influenced by the stress state of the material. 

A common way of evaluating the susceptibility of a given material to SCC processes is by testing cracked rods under a constant extension rate tensile (CERT) test in a harsh environment [3]. A crack is generated by an initial sharp notch by applying cyclic loading (*fatigue precracking*) with diverse loading sequences [8] exceeding the material fatigue crack propagation threshold in terms of the stress intensity factor (SIF) amplitude (Δ*K*_th_). As a result of the precracking process, residual stress field appears in the vicinity of the crack tip (CT), depending on the maximum cyclic SIF level (*K*_max_) applied during the final stage of fatigue precracking. This residual stress state influences hydrogen diffusion and hence, the hydrogen accumulation and microstructural damage causing HAC. An adequate way of obtaining the hydrogen distribution near CT is by numerical simulation of stress-assisted hydrogen diffusion in the material [9,10]. 

In this paper, numerical simulations were performed to establish the role of the residual stresses generated after fatigue precracking, considering different *K*_max_-levels. The residual stress states and the stress re-distributions during CERT test in the CT vicinity were obtained by the numerical simulations of the fatigue precracking and posterior CERT loading of a cracked pearlitic steel rod by using a commercial finite element (FE) analysis code. Later, from the previously computed stress states, the hydrogen accumulation in the CT vicinity was obtained by simulating the stress-assisted diffusion of hydrogen using an *in-house* FE code. The results provide key information with regard to the HAC process for a better understanding of the role of fatigue precracking in HAC. 

## 2. Numerical Modelling

The modelling that was undertaken consists in the simulation of hydrogen diffusion in CERT experiments that have been described elsewhere [11]. The analysis was divided into two sequential numerical simulations. The first one (*mechanical*) obtains the stress state, including the residual stress state after fatigue precracking and its evolution during the CERT test. The second one (*stress-assisted hydrogen diffusion*) determines the evolution with time of the hydrogen accumulation in a material assisted by the previously computed mechanical stress states. 

**Figure 1:**
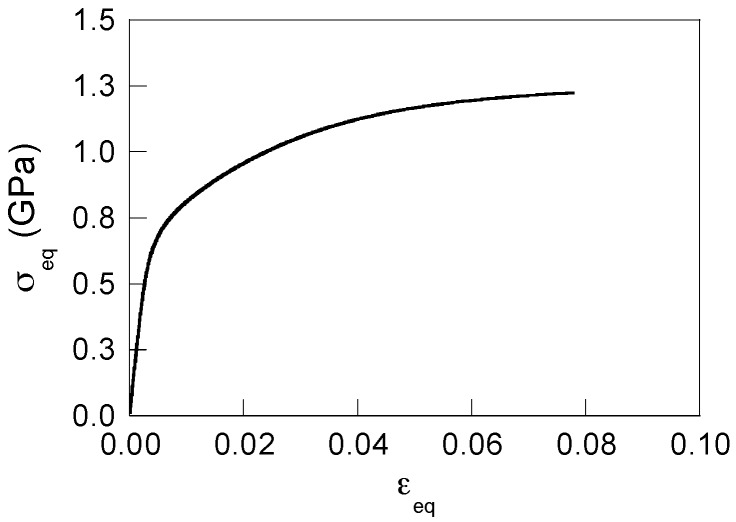
Experimental curve (equivalent stress, *σ*_eq_ versus equivalent strain, *ε*_eq_) for the studied hot-rolled pearlitic steel.

The material used in this study is a pearlitic steel rod of diameter *d* = 11.03 mm, its chemical composition is as follows: C 0.789%, Mn 0.681%, Si 0.210%, P 0.010%, S 0.008%, Al 0.003%, Cr 0.218%, V 0.061%. The principal mechanical characteristics of the steel (Young modulus *E*, yield strength *σ*_Y_, ultimate tensile stress (UTS) *σ*_R_) obtained from the corresponding experimental master curve shown in Figure 1 are summarized in Table 1. The material fracture toughness (*K*_IC_) was taken from a previous study [12] dealing with this pearlitic steel. 

A common experimental technique [11,13,14,15] of fatigue precracking consists of applying a multi-step cyclic loading with a progressively decreasing maximum load level (*K*_max_) in various steps. In this procedure, the maximum loading of the cycling decreases as the number of precracking step increases. Once the crack is originated by the initial fatigue loading step with the largest load level, crack growth continues under a lower fatigue loading until the effects of the plastic zone generated by the earlier step are considered to be removed. In this way, precracked samples with the same crack length *a* = 0.3*d* were obtained in the SCC experiments [11], which are simulated in this work. They had different plastic zones and residual stresses depending on the *K*_max_ used in the last fatigue precracking step.

**Figure 2:**
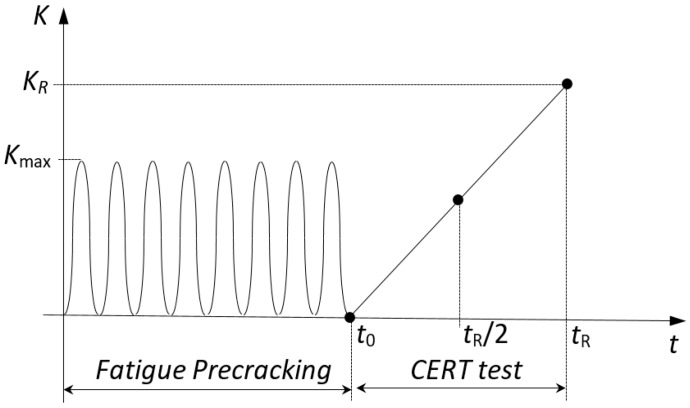
Scheme of the loading history in terms of the stress intensity factor (SIF) *K* during the process of fatigue precracking and posterior monotonic loading during the constant extension rate tension (CERT) test. *K*_R_ represents the critical SIF at final fracture.

In this study, the simulations of the described fatigue precracking of the CERT test specimens were limited to the final stage of the real precracking process, because this is the stage that is responsible for the residual stress fields in the specimens before the SCC tests. Different schemes of precracking by cyclic loading were considered. All of them have sinusoidal shape varying from a null load (*K*_min_ = 0) to a maximum load (*K*_max_), with a load ratio *R_K_* = *K*_min_/*K*_max_ = 0 (Figure 2). The difference between the fatigue (cyclic) loading schemes is the value of *K*_max,_ which was determined by the values used in the simulated experiments [11]. The four *K*_max_ levels expressed in terms of the fractions of the material´s fracture toughness (*K*_IC_) are as follows: (i) the heavy fatigue precracking, Loading regime I (*K*_max_ = 0.80*K*_IC_), two moderate fatigue precracking regimes, (ii) Loading II (*K*_max_ = 0.60*K*_IC_) and (iii) Loading III (*K*_max_ = 0.40*K*_IC_), and finally, (iv) the soft fatigue precracking, Loading IV (*K*_max_ = 0.25*K*_IC_). 

After fatigue precracking, a monotonically rising loading up to the final fracture is considered to be aiming to reproduce the conditions of the modelled CERT tests performed for estimating the SCC susceptibility of the steel [11]. The specimens during CERT tests were loaded under controlled displacement rate conditions, and the employed displacement rate was adjusted to render a constant overall deformation rate as low as 0.01 mm·min^−1^, which is in the range of slow loading that provides sufficient time for hydrogen diffusion, and consequently, for HE to occur. The experimentally obtained [11] fracture loads and fracture times for different tests are presented in Table 2. It can be deduced that in terms of the applied load *F* the modelled CERT test conditions correspond to the loading rate *dF*/*dt* = 0.002 kN/s. 

To carry out the first stage of the simulations (*mechanical analysis*) adequately and obtain reliable high-resolution data about the stress-strain fields in the close proximity of the CT, two nonlinearities must be considered: the physical one to account for the elastoplastic material behavior, and the geometrical one to account for large deformations and strains, which arise near the CT [16]. With regard to the first, the constitutive model of elastoplastic material with the isotropic hardening rule and von Mises yield criterion was adopted in this research, where the employed master curve “equivalent stress-equivalent strain”, given in the plot of Figure 1, replicated the experimental stress-strain curve of the steel. 

This modelling approach does not take into account the potential effects of hydrogen on the stress-strain constitutive relation of the steel, or on the stress-strain fields in hydrogenated metal. The reasons are summarized in the following sentences. Firstly, the effects of hydrogen on the stress-strain behaviors of steels are uncertain: numerous reported data [17,18] have manifested slight both strengthening and softening influences of hydrogen in steels (increase and decrease of the yield strength), but the alterations of the stress-strain curve shapes by hydrogen were rather minor (within few percent of yield strength). Accordingly, this factor is considered to be insignificant for the CT stress-strain fields. Secondly, possible generation of strains, and subsequent stresses in metals because of the crystal lattice expansion by interstitial hydrogen is regarded as insignificant in this study. Indeed, according to available evaluations [19], the introduction of hydrogen into BCC iron lattice can generate stresses as high as about 2 MPa per 10 mol/m^3^ (1.2 ppm by mass) of hydrogen concentration in steel. So, hydrogenation attainable near the CT in steels during SCC, which usually does not exceed ~5 ppm, cannot cause a substantial effect on the mechanical fields which have stress levels exceeding the magnitudes of ~10^3^ MPa. 

Numerical simulations of the loading schemes including indicated fatigue precracking regimes and posterior monotonic loading up to the final fracture (Figure 2) were carried out in this way. Regarding the precracking loading, the Laird and Smith mechanism of the fatigue crack growth by blunting-resharpening [16,20], which is considered suitable for analysing the cracking phenomena in ductile materials, relies on the near-tip plastic deformations under cyclic loading with no bond breaking. Accordingly, high-resolution large-deformation elastoplastic simulation of the crack is the right way to visualize the fatigue crack growth [21,22]. In the numerical simulation of fatigue precracking, the modelling of cyclic crack growth only considers the elastoplastic crack advance according to the mechanism of Laird and Smith, but not by bond breaking. 

**Figure 3:**
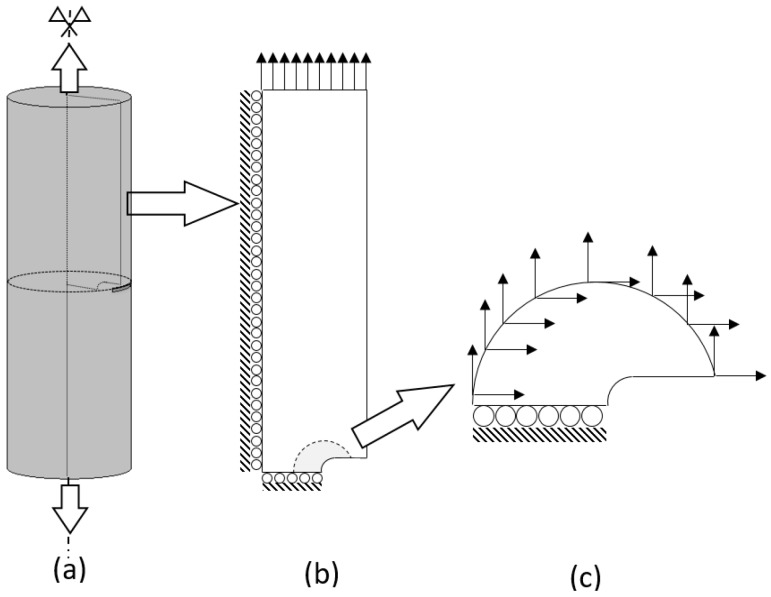
Simplification of the 3D cracked rod into a two-dimensional model and the *K*-dominance region in the vicinity of the crack tip (CT): (**a**) 3D geometry; (**b**) bi-dimensional simplified model and (**c**) *K*-dominance region and depicted nodal displacements applied according to the boundary–layer approach.

The geometry of the analysis case is given in Figure 3a: a cracked rod with a semi-elliptical crack placed in the rod on the central cross section normal to its axis. This geometry has no revolute symmetry and hence, the axisymmetric formulation is not suitable. However, the 3D geometry can be simplified to a bi-dimensional plane strain case accounting for the constraint effect that arises near the crack front in the central longitudinal section of the rod, as shown in Figure 3b. In this way, computing time can be saved in both simulations. 

In this scheme, the parallel-flanks slot of the width 2*b*_0_ with semi-circular tip was taken as a model of an undeformed crack (Figure 3c), as it has been repeatedly substantiated and widely used elsewhere [23,24,25,26,27,28]. During axial loading, the points placed at the rod axis are fixed in the radial direction, and the points at the central cross section (where the crack is situated) are fixed in the axial direction due to the symmetry. Taking these conditions into account, the geometry can be simplified to a quarter, as shown in Figure 3b. This geometry was non-uniformly meshed with 4-node quadrilateral elements. The sizes of the elements were progressively increased as the distance from the CT grew. Thus, a fine mesh was generated near the CT, whereas a coarse mesh was applied away from the CT (remote zone). 

The previously described loads (Figure 2) were applied as imposed displacements at the top edge of the two-dimensional sample used in computations (Figure 3b) [20]. The large number of FE nodes used in this model made it unsuitable for the later simulations of hydrogen diffusion assisted by stress due to excessive computing time. To reduce the computational expenses, the so-called *boundary layer approach* [16,20], which has been commonly used under monotonic loading [25] and employed for cyclic one [29], was invoked as it is schematised in Figure 3c. Here, *K*-controlled boundary conditions given by the known singular linear-elastic solution for the near-tip displacements *u_x_* and *u_y_* were imposed over a remote boundary of a circular domain of a radius sufficiently large in comparison to the CT plastic zone size *r*_p_. 

**Figure 4:**
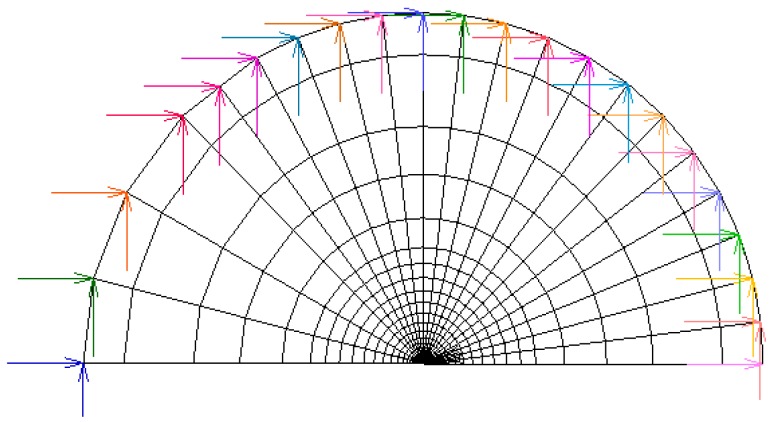
View of the mesh associated with the *K*-dominated region used in the numerical analysis following the boundary layer approach.

This approach is based on the well-known similitude principle of fracture mechanics [16,22,25,26,27]: stress-strain states near the CT only depend on SIF. Therefore, the analysis can be limited to the *K*-dominated zone placed next to the CT with boundary conditions according to the fundamental elastic solution given by Muskhelishvili [30]. In order to achieve substantial computer time saving, a simplified case was considered in terms of *K*-dominated zone by using the nearby CT mesh (the shadowed zone as schematised in Figure 3 and displayed in detail in Figure 4).

One of the conditions that must be fulfilled to ensure the validity of this approach is that the size of the *K*-dominated zone must be large enough enabling to consider negligible the perturbation of the *K*-dominated field by the process zone, where plastic strains are generated near the CT, and in addition, small enough in relation to the remote zone (far from the effects of the crack) to make negligible the perturbation of the *K*-dominance by this non-singular far-field. In addition, to ensure the *K*-dominance of the near-tip elastoplastic fields in the employed model of a crack [16], the crack tip opening displacement (CTOD) *δ*_t_ must overcome the value given by [16,30]:

(1) $$\delta_{t}=0.6\frac{K^{2}}{E\sigma_{Y}}\geq 2b_{0}$$

*b*_0_ being the CT half-width.

With regard to the dimensions of the *K*-dominated zone, an estimation of the plastic zone size (*r*_p_) is necessary. It is estimated as follows [31,32,33].

(2) $$r_{p}=0.15\frac{{\bar{K}}_{\max}^{2}}{{\left(\frac{\sigma_{Y}+\sigma_{R}}{2}\right)}^{2}}$$

where ${\bar{K}}_{\max}$ is the upper bound of attainable SIF values and (*σ*_Y_ + *σ*_R_)/2 is employed instead of the habitual *σ*_Y_ to account approximately for the strain hardening. 

The fatigue precracking loading (Figure 2) was applied by means of imposed displacements at nodes (*u_x__,i_*, *u_y__,i_*) placed in the periphery of the optimal *K*-dominated zone as shown in Figure 4. For the simulation of the monotonic loading, a fixed *K* rate (*dK*/*dt*) of 0.078 MPa·m^1/2^/s was applied up to final fracture, which corresponds to the experimental loading rate according to the data in Table 2. These displacements imposed to each node were calculated on the basis of the adopted boundary layer approach with the use of the known SIF solution for the edge-crack plates [16]. 

Once the stress state is revealed from the results of the first simulation of the CT mechanics, the second numerical simulation of the stress-assisted diffusion of hydrogen can be performed to obtain the hydrogen concentrations for diverse times of exposure to the hydrogenating source. As previously discussed, the stage that rules HAC, HAF or HE phenomena in pearlitic steels is hydrogen transport in the material by diffusion [34,35,36] dependent on the stress state [36,37,38,39,40].

In numerical simulations, the model for hydrogen diffusion assisted by stress is implemented [37,38]. Briefly, in this model the hydrogen flux (***J***) can be expressed by the modified Fick law including the influence of stresses, as follows [37,38]:

(3) $$J=D\left\{-\nabla C+C\left(\frac{v_{H}}{RT}\nabla \sigma \right)\right\}$$

*D* being the hydrogen diffusion coefficient, *C* the hydrogen concentration, *v*_H_ the partial molar volume of hydrogen in metal, *R* the universal gas constant and *T* the absolute temperature. 

Applying the mass conservation law [41], the evolution of hydrogen concentration with time can be represented by the following Equation [37,38].

(4) $$\frac{\partial C}{\partial t}=-\nabla \cdot \left[-D\nabla C+DC\left(\frac{v_{H}}{RT}\nabla \sigma \right)\right]$$

This differential equation cannot be analytically solved and, consequently, a numerical solution is necessary. When the system metal-environment is in equilibrium, i.e., ***J*** = 0, the equilibrium hydrogen distribution in stressed metal is given by the following expression [28,37,38] for the equilibrium concentration (*C*_eq_):

(5) $$C_{\mathrm{eq}}=C_{0}\exp\left[\frac{v_{H}}{RT}\sigma \right]$$

where *C*_0_ is the hydrogen equilibrium concentration in the metal free of stress. 

This formula represents the equilibrium distribution of hydrogen in the metal-environment system which gives the maximal attainable hydrogen concentration in stressed metal. This equilibrium hydrogen distribution coincides with the steady state solution of the diffusion Equation (4) at time *t* → ∞ for the specimen immersed in the hydrogenating environment and subjected to sustained load. Assuming quick mass exchange between the metal and the environment on the hydrogen entry surface, the equilibrium concentration value given by the expression (5) may be used as a boundary condition for hydrogen diffusion in metal [28,37,38].

An FE modelling code developed *ad-hoc* was used for numerical simulation of the hydrogen diffusion assisted by stress. The weak form of the weighted residuals approach is employed considering the Galerkin method for approximating the stress distribution via FE nodal functions *N_i_* [42]. As a result, the following FE equation system is obtained:

(6) $$\begin{array}{r} \sum_{j}\left[-\int_{V}N_{i}N_{j}dV\right]\frac{d\left[C\right]}{dt}+\sum_{j}[\int_{V}\left[D\nabla N_{i}-D\frac{v_{H}}{RT}(\nabla \sigma \nabla N_{i})N_{j}\right]dV+\vartheta \int_{S_{\mathrm{eq}}}N_{i}N_{j}dS]C_{j}=\\ =J_{f}\int_{S_{f}}N_{i}dS+\vartheta C_{\mathrm{eq}}\int_{S_{\mathrm{eq}}}N_{i}dS\;i=1...n \end{array}$$

*S*_eq_ is the surface exposed to hydrogenation, *ϑ* is a constant representing the mass exchange rate at this surface, and *n* is the number of FE nodes. This equation system can be expressed in a matrix reduced form as follows:

(7) $$\left[M\right]\frac{d\left[C\right]}{dt}+\left[K\right]\left[C\right]=\left[F\right]$$

The algorithm stated by Zienkiewicz et al. [42] was used for solving the system (7) of linear ordinary differential equations with respect to the nodal concentration values [*C*]:

(8) $$\left({\left[C\right]|}_{q}-{\left[C\right]|}_{q-1}\right)\frac{\left[M\right]+\tau \Delta t\left[K\right]}{\Delta t}+\left[K\right]{\left[C\right]|}_{q-1}=\left[F\right]$$

where the time increment Δ*t = t*_q_ − *t*_q−1_, and the constant *τ* are chosen in such a way that the stability of this algorithm is ensured. Within the present work the algorithm is unconditionally stable for values *τ* ∈ [0.5,1].

The described FE approach was employed considering the two-dimensional case shown in Figure 4 using four-node quadrilateral elements. The same mesh was used both in the *mechanical* simulation and in the analysis of hydrogen diffusion assisted by stress. For all the cases of this study, the required convergence of results was achieved in simulations with the selected meshes.

## 3. Residual Stress Distributions in Fatigue Precracked Rods

**Figure 5:**
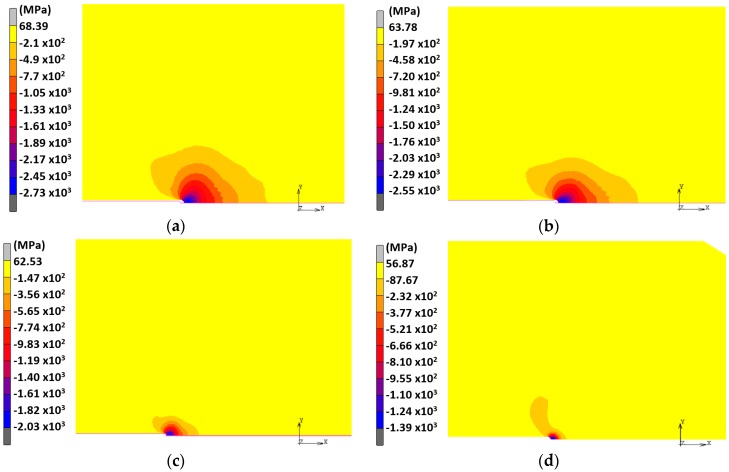
Hydrostatic stress fields in the vicinity of the CT obtained at the end of the fatigue precracking at various *K*_max_: (**a**) 0.80*K*_IC_; (**b**) 0.60*K*_IC_; (**c**) 0.40*K*_IC_; (**d**) 0.25*K*_IC_.

According to the previously described model of hydrogen diffusion assisted by stress [37,38], special attention must be paid to the hydrostatic stress since the gradient of this variable is one of the driving forces of hydrogen diffusion. For this reason, the distributions of hydrostatic stress for each case of study were analysed at different times during CERT loading. Figure 5 shows the hydrostatic stress fields in the CT vicinity obtained at the end of the fatigue precracking loading (*residual stress state*) for each case of study, which is the initial stress state at the beginning of the CERT tests. 

According to these results, such a field is localized in the vicinity of the CT, reaching progressively smaller absolute values of the stress *σ* as the maximum fatigue precracking levels *K*_max_ decrease. Taking this into account, the analysis is focused on the distribution of this variable in the central cross section of the rod, which contains the crack, at three different instants during the CERT loading: (i) the initial residual stress state as a consequence of the cyclic precracking before CERT; (ii) the intermediate time during the CERT and finally; (iii) the instant *t* = *t*_R_ corresponding to the final fracture of the sample under HAC conditions at *K* = *K*_R_ known from the simulated experiments [11] (Table 2). 

**Figure 6:**
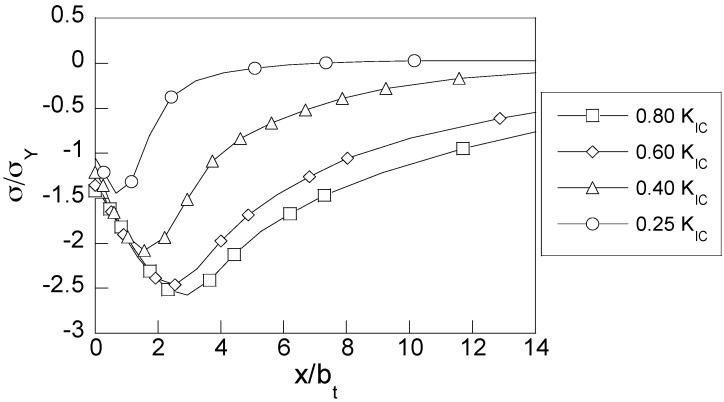
Distributions of the dimensionless *residual hydrostatic stress* near the CT for rods subjected to different fatigue precracking regimes against the dimensionless distance from the CT.

In the case of the residual stresses generated after precracking, Figure 6 shows the distributions of the dimensionless hydrostatic stress (*σ*/*σ*_Y_) against the dimensionless distance to the CT, *x*/*b*_t_, *b*_t_ being the deformed CT width at the end of precracking, *b*_t_(*K*_max_) = *b*_0_ + *δ*_t_(*K*_max_), which is different for each *K*_max_ level according to Equation (1).

Two effects produced by the applied precracking are revealed here; (i) the maximum value of the compressive residual stress (−*σ*) is higher as the precracking level is increased and (ii) the position of the extreme value of the hydrostatic stress *σ* is shifted further from the CT into the metal depth. In addition, it is worth emphasizing that slight differences of the boundary values of the hydrostatic stress are observed just at the CT for different fatigue loading levels, which are notably higher than the material yield strength, indicating the important role of plasticity in the fatigue phenomena.

With regard to different distributions of the residual hydrostatic stress associated with distinct fatigue precracking levels, the main differences are found at a certain distance from the CT, where the extreme of the stress is located. This fact makes the *gradients of hydrostatic stress* (a driving force for hydrogen diffusion according to the employed model) similar for all cases of study. This is consistent with previous studies that found a beneficial effect of these compressive residual stresses in environmental damage phenomena such as HAC [43,44]. 

**Figure 7:**
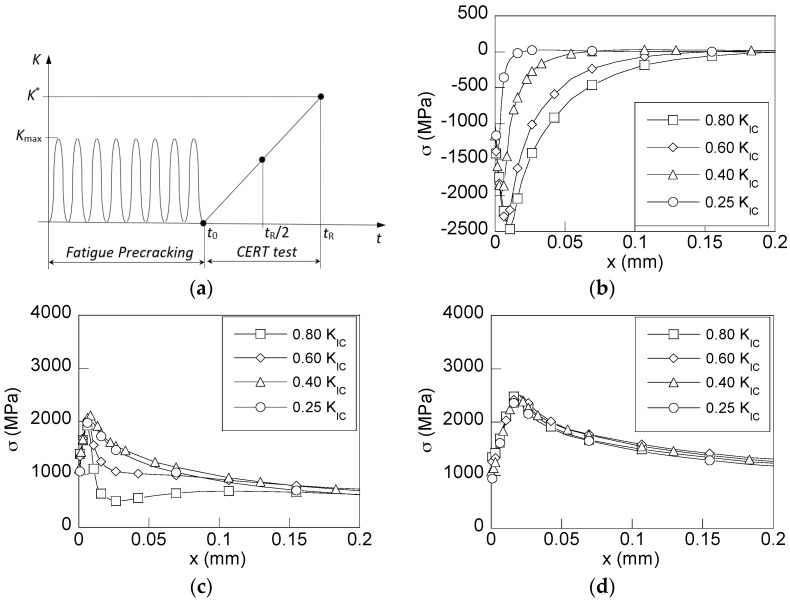
(**a**) Scheme of the fatigue precracking, CERT loading and the distributions of hydrostatic stress in the CT vicinity in rods subjected to different fatigue precracking *K*_max_-levels, represented as a function of the distance from the CT *x*, at different times during the hydrogen assisted cracking (HAC) process: (**b**) initial; (**c**) intermediate (*t*_R_/2); (**d**) final fracture (*t*_R_).

Furthermore, the evolution of the hydrostatic stress distribution during constant rate monotonic loading after precracking is shown in Figure 7 for three different instants of time during the CERT test: the initial, associated with the residual stress field, the intermediate time (*t*_R_/2), and the instant of final fracture by HAC (*t*_R_), where *t*_R_ is given in Table 2 [11]. According to these results, the fracture time is increased with the level of fatigue precracking load applied to the sample.

During CERT tests, the applied loading generates a tensile stress state which progressively annihilates compressive stresses generated after fatigue precracking. It is evident that this process takes more time for those precracking loads that cause higher compressive stresses and hence, the effects of the previous compressive residual stresses disappear at higher loads during the CERT test. This effect is clearly seen in Figure 7c where the residual stress distributions generated by low precracking loads have no appreciable effect on the stress state under posterior rising load, whereas for the heavy precracking regimes the effects still remain in the stress distributions under a rising load during HAC test.

At the critical instant *t*_R,_ which corresponds to the final fracture at HAC according to the experiments (see Table 2), the effect of the compressive residual stress is completely removed by the CERT load for all considered precracking *K*_max_ levels, see Figure 7d. The critical distributions of stress shown in Figure 7d are obtained for distinct times of exposure to the aggressive environment (fracture times, *t*_R_ presented in Table 2) depending on the applied fatigue precracking load. Taking into account that the stress distributions at the fracture instants for all the regimes are quite similar for all the precracking loads, it can be determined that this corresponding distribution is associated with a critical stress field leading to final fracture by HAC mechanism. However, it is necessary to point out that different levels of fatigue precracking and, *per ende*, different initial compressive states, determine different values for the fracture time for each case of study. 

## 4. Hydrogen Distributions in Fatigue Precracked Rods

**Figure 8:**
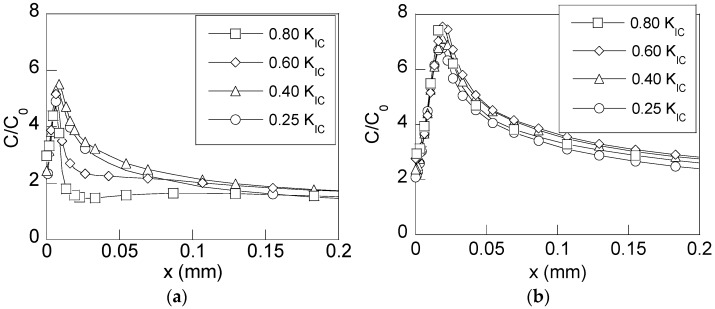
Distributions of the hydrogen concentration in the CT vicinity in rods subjected to different fatigue precracking *K*_max_-levels against the distance from the CT at diverse times during HAC process: (**a**) the intermediate time, *t* = *t*_R_/2; (**b**) the final time, *t* = *t*_R_.

From the stress states that appear in the simulated samples after the precracking process and their subsequent loading during CERT tests in cathodic conditions, the hydrogen accumulation was obtained by means of numerical simulation of the hydrogen diffusion assisted by stress. Figure 8 shows the distributions of the relative hydrogen concentration (*C*/*C*_0_) against the depth from the CT in the transversal section of the rods precracked by fatigue, which are obtained at two instants of the CERT test in cathodic environment: (i) the intermediate instant (*t*_R_/2), and the final fracture instant (*t*_R_).

According to the results shown in Figure 8, the samples undergoing more intense precracking loadings (higher *K*_max_-levels), and consequently, those that have higher compressive residual stresses in the near-tip area, have a lower concentration of hydrogen in the CT vicinity as a consequence of stress-driven hydrogen diffusion. This way, the beneficial effect of compressive stresses is revealed since they produce a delay in hydrogen accumulation near the CT, and consequently, a delay in the microstructural hydrogen induced damage, thereby leading to longer times of fracture under cathodic environments promoting HAC. However, once the earlier-created residual stress state is passed, and consequently, the effect of compressive stresses is completely removed, the hydrogen distributions obtained for each precracking loading are similar (Figure 8b). From this result, the critical distribution of hydrogen concentration linked to the time of fracture caused by HAC is revealed.

**Figure 9:**
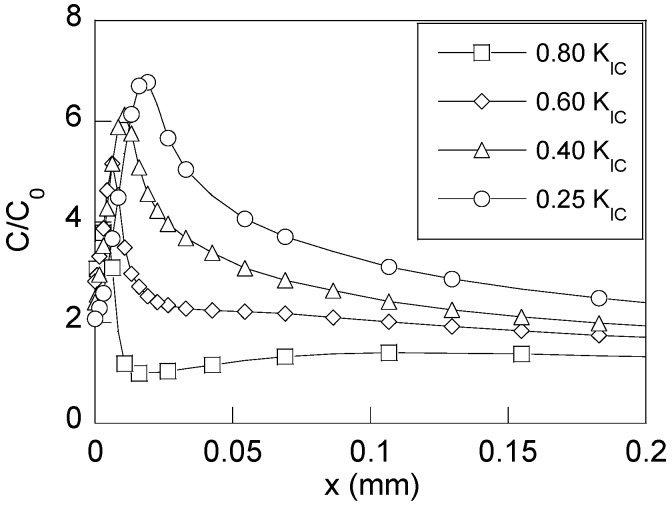
Distributions of the hydrogen concentration for a time equal to the fracture time of the rod precracked with *K*_max_ = 0.25*K*_IC_ for the four levels of *K*_max_ used in the CERT tests under HAC conditions (*K*_max_ = 0.80*K*_IC_, 0.60*K*_IC_, 0.40*K*_IC_ and 0.25*K*_IC_).

Finally, for revealing the effect of the compressive stresses generated after fatigue precracking, the hydrogen distributions near the CT were obtained for all the cases of study at fixed time of exposure to the hydrogenating source. Figure 9 shows the distributions of hydrogen concentration for four analysed cases at the time corresponding to the fracture instant associated with the lowest fatigue precracking regime of *K*_max_ = 0.25*K*_IC_, i.e., at *t*_R_ = 17,000 s (Table 2). 

The observed *barrier effect* exerted by the compressive residual stress field increases with the fatigue precracking level (*K*_max_) as it is clearly shown by the presented hydrogen concentration distributions for the two higher precracking levels of 0.80*K*_IC_ and 0.60*K*_IC_. In the other two cases, the compressive residual stress effect is cancelled by CERT loading for this time of exposure, and consequently, this effect on the SCC process by HAC vanishes. As soon as the effects of the residual stresses are cancelled, i.e., when the precracking level is lower, the microstructural damage caused by hydrogen will begin.

## 5. Conclusions

The compressive residual stress distribution generated in a pearlitic steel rod after fatigue precracking procedure before testing the hydrogen assisted cracking (HAC) susceptibility of steels plays a key role in hydrogen accumulation, and hence, in microstructural hydrogen damage. Such compressive residual stresses are produced by strain compatibility near the CT.

According to the obtained results, as the maximum fatigue precracking level (*K*_max_) is increased, the compressive residual stress is higher. In addition, the extreme value of this residual stress distribution is further away from the CT. This creates the *barrier effect* against hydrogen diffusion, which extends over a wider zone. 

The beneficial effects of the compressive residual stresses generated by fatigue precracking are cancelled when a stress state equivalent to the one applied by fatigue precracking is reached during the constant extension rate tension (CERT) test. Thus, the higher the *K*_max_, the longer the time needed for cancelling the barrier effect at a given loading rate.

The distributions of hydrogen concentration at the experimentally obtained fracture times (when the compressive residual stresses are fully cancelled for all the cases of study) are the same for all *K*_max_-levels considered in the analysis. This distribution is linked to a critical hydrogen accumulation causing HAC fracture in the rod. 

## Figures and Tables

**Table 1:** Mechanical properties of the hot-rolled pearlitic steel.

*E* (GPa)	*σ*_Y_ (GPa)	*σ*_R_ (GPa)	*K*_IC_ (MPa·m^1/2^)
202	0.696	1.224	52.8

**Table 2:** Experimental fracture loads and fracture times of CERT tests of specimens precracked by fatigue at various levels of *K*_max_ [11].

*K*_max_	*t*_R_ (s)	*F*_R_ (kN)
0.80*K*_IC_	22,900	48.1
0.60*K*_IC_	21,600	44.0
0.40*K*_IC_	21,000	38.3
0.25*K*_IC_	17,000	31.3

## Abbreviations

The following abbreviations are used in this manuscript:

BCC	Body centered cubic
CERT	Constant extension rate tension
CT	Crack tip
CTOD	Crack tip opening displacement
FE	Finite element
HAC	Hydrogen assisted cracking
HAF	Hydrogen assisted fracture
HE	Hydrogen embrittlement
LAD	Localized anodic dissolution
SCC	Stress corrosion cracking
SIF	Stress intensity factor
UTS	Ultimate tensile stress
*a*	Crack length
*b*_0_	Crack tip initial half-width
*b*_t_	Current (deformed) crack tip half-width
[*C*]	Vector of the FE nodal values of hydrogen concentration
*C*	Hydrogen concentration
*C*_0_	Hydrogen concentration in the metal free of stress in equilibrium with hydrogenating environment
*C*_eq_	Hydrogen concentration in equilibrium state in stressed metal
*d*	Specimen diameter
*D*	Hydrogen diffusion coefficient
*E*	Young modulus
[*F*]	Assembled FE boundary condition matrix
*i*	Counter
***J***	Hydrogen flux vector
***J***_f_	Hydrogen flux on the surface *S*_f_
*K*	Stress intensity factor (SIF)
*K*_IC_	Fracture toughness
*K*_max_	Maximum SIF at cyclic loading
*K*_min_	Minimum SIF at cyclic loading
*K*_R_	Critical SIF at CERT test final fracture
[*K*]	Assembled FE diffusivity matrix
[*M*]	Assembled FE capacity matrix
*n*	Number of FE nodes
*N_i_*	FE nodal functions
*r*_p_	Characteristic size of the plastic zone
*R*	Universal gas constant
*R_K_*	Load ratio
*S*	Specimen surface
*S*_eq_	Specimen surface exposed to the hydrogenating source
*S*_f_	Specimen surface with a flux boundary condition imposed
*t*	Time
*t*_q_	Time corresponding to the increment *q* in numerical integration
*t*_R_	Final fracture time by hydrogen assisted cracking
*T*	Absolute temperature
*u_x__,i_*	Deformation displacement of node *i* in *x* axis direction
*u_y__,i_*	Deformation displacement of node *i* in *y* axis direction
*v*_H_	Partial molar volume of hydrogen in metal
*V*	Volume
*x*	Cartesian coordinate along the crack plane originating at the CT
*y*	Cartesian coordinate normal to the crack plane originating at the CT
*δ*_t_	Crack tip opening displacement (CTOD)
Δ*K*	Amplitude of the stress intensity factor (SIF) during fatigue preloading
Δ*K*_th_	Fatigue crack propagation threshold
*ε*_eq_	Equivalent strain
*ϑ*	Mass exchange constant on metal-environment surface
*σ*	Hydrostatic stress
*σ*_eq_	Equivalent stress
*σ*_R_	Ultimate tensile stress (UTS)
*σ*_Y_	Yield strength
*τ*	Stability factor in numerical integration scheme
